# 2661. Characterization of Mpox in Southeast Michigan

**DOI:** 10.1093/ofid/ofad500.2272

**Published:** 2023-11-27

**Authors:** Rija B R Alvi, Yasmeen Mann, Simran Brar, Indira Brar, Geehan Suleyman

**Affiliations:** Henry Ford Hospital, Detroit, Michigan; Henry Ford Hospital, Detroit, Michigan; Wayne State School of Medicine, Detroit, Michigan; Henry Ford Hospital, Detroit, Michigan; Henry Ford Health, Detroit, Michigan

## Abstract

**Background:**

More than 30,000 cases of mpox have been identified in the United States. Data suggest that about 40% of affected persons are co-infected with HIV. Although most cases are self-limiting, patients with low CD4 counts are at increased risk of developing severe clinical manifestations and dying.

**Methods:**

Retrospective case-control study comparing mpox cases among people with HIV (PWH) and HIV-uninfected persons from July to Dec 2022, at Henry Ford Health in Southeast Michigan. Demographic data, clinical characteristics, treatment, and outcomes were evaluated.

**Results:**

54 patients were diagnosed with mpox. Overall, 42 (78%) were MSM or bisexual men, 36 (67%) Black, and 34 (63%) PWH; median age was 34.5 years (Table 1). The majority (63%) had prior sexually transmitted infections (STIs), which were more common in PWH (82% vs 30%, p< 0.001). About one-third had multiple sexual partners; more than half engaged in insertive or receptive anal intercourse. 32 (94%) of PWH were on ART, 26 (76%) had CD4 counts >200 and 19 (60%) had undetectable viral load; mean CD4 count was 602 and viral load 7942. Of the HIV-uninfected persons, 5 (25%) were on PrEP. Receipt of mpox vaccine was uncommon in either group. All patients presented with rash that was disseminated in 35%. Fever/chills, lymphadenopathy, headache, proctitis and pharyngitis were the most common manifestations and did not differ among the two groups. Concomitant STIs were present in 22 (48%) of 46 persons tested; syphilis co-infection was more prevalent among PWH (35% vs 25%, p< 0.006). Hospitalization and receipt of tecovirimat were similar between the two groups; no patients died.

Demographics, Risk Factors, Clinical Manifestations and Outcomes of Mpox Patients

**Figure:**
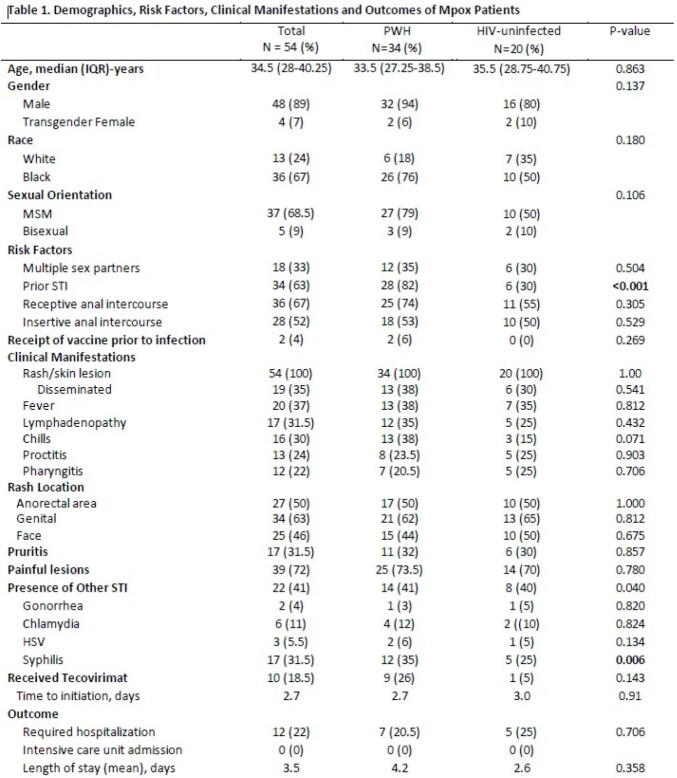

**Conclusion:**

Overall, there were no significant differences in clinical manifestations or outcomes between PWH and HIV-uninfected persons with mpox except for syphilis co-infection. Most of our PWH cohort was on ART and virally suppressed with high CD4 count. Hence, efforts should focus on rapid treatment of PWH with effective ART to achieve virological suppression and immunological recovery to minimize clinical complications and severe outcomes associated with opportunistic pathogens. Vaccinating all high-risk individuals, early mpox recognition and testing, and screening for additional STIs should be prioritized.

**Disclosures:**

**Indira Brar, MD**, Gilead: Advisor/Consultant|Gilead: Grant/Research Support|Gilead: Honoraria|Janssen: Grant/Research Support|Janssen: Honoraria|ViiV: Advisor/Consultant|ViiV: Grant/Research Support|ViiV: Honoraria

